# Controversies in ureteroscopy: lasers, scopes, ureteral access sheaths, practice patterns and beyond

**DOI:** 10.3389/fsurg.2023.1274583

**Published:** 2023-09-13

**Authors:** Patrick Juliebø-Jones, Etienne Xavier Keller, Vincent De Coninck, Sabine Uguzova, Lazaros Tzelves, Mathias Sørstrand Æsøy, Christian Beisland, Bhaskar K. Somani, Øyvind Ulvik

**Affiliations:** ^1^ Department of Urology, Haukeland University Hospital, Bergen, Norway; ^2^ Department of Clinical Medicine, University of Bergen, Bergen, Norway; ^3^ EAU Young Academic urology Urolithiasis Group, Arnhem, Netherlands; ^4^ Department of Urology, University Hospital Zurich, University of Zurich, Zurich, Switzerland; ^5^ Department of Urology, AZ Klina, Brasschaat, Belgium; ^6^ Department of Urology, Royal Preston Hospital, Preston, United Kingdom; ^7^ 2nd Department of Urology, Sismanogleion Hospital, National and Kapodistrian University of Athens, Athens, Greece; ^8^ Department of Urology, Southampton General Hospital, Southampton, United Kingdom

**Keywords:** ureteroscopy, ureteral access sheath, fluroscopy, laser, lithotripsy

## Abstract

Ureteroscopy has become an increasingly popular surgical intervention for conditions such as urinary stone disease. As new technologies and techniques become available, debate regarding their proper use has risen. This includes the role of single use ureteroscopes, optimal laser for stone lithotripsy, basketing versus dusting, the impact of ureteral access sheath, the need for safety guidewire, fluoroscopy free URS, imaging and follow up practices are all areas which have generated a lot of debate. This review serves to evaluate each of these issues and provide a balanced conclusion to guide the clinician in their practice.

## Introduction

Ureteroscopy (URS) stands beside shockwave lithotripsy (SWL) and percutaneous nephrolithotomy (PCNL) in the trilogy of minimally invasive therapies performed in patients with urolithiasis. Since the early descriptions by Marshall et al. in 1964, it has undergone many modifications, both in terms of the equipment used as well as the surgical techniques applied (1–3). These advances have elevated the position of URS to that of a treatment of choice in a wide variety of clinical scenarios and complex stones, and the patient selection has expanded accordingly. To this end, URS has been demonstrated in clinical studies to be not only safe but also a preferred option in certain situations such as pregnancy, patients at the extreme of age and stones in the lower pole (4–8). The abovementioned modifications include technological advancements such as the energy sources employed for stone lithotripsy, the development of digital and single use ureteroscopes as well as novel accessories such as anti-retropulsion devices and more recently, real time intra-renal pressure monitoring systems (9). While it is a luxury for the modern-day surgeon to have such an array of technologies at their disposal, which have certainly contributed to the improved outcomes associated with URS, it has led to a wide range of practice patterns as well as ongoing debate regarding the actual advantages and disadvantages they may actually yield for the patient (10). While there is an increasing volume of studies which have sought to evaluate these individual areas, it can be difficult for the time pressured clinician to maintain a balanced and informed viewpoint on these areas of debate and controversy. Our aim was therefore to review the literature and deliver such information.

## Methods

A non-systematic literature review was performed in order to gain evidence to allow for evaluation of the following key topics: Role of single use ureteroscopes, optimal laser for stone lithotripsy, basketing versus dusting, the impact of ureteral access sheath (UAS), the need for safety guidewire (SGW), fluoroscopy free URS, imaging and follow up practices are all areas which have generated a lot of debate over the last few years and are reviewed in the present study. Each of the specific words were used as search terms. Bibliographic databases searched included Medline, Google Scholar and the Cochrane Library. Only articles in the English language were assessed but all article types were included. The findings have been presented in a narrative format.

### Single use ureteroscopes

Until recently, all flexible ureteroscopes have been reusable. In October 2015, the first fully disposable single use (SU) and commercially available flexible ureteroscope was introduced (Lithovue™, Boston Scientific, Marlborough, MA). Approximately 30 models are now available from different companies. Reducing the infection and contamination risk, potential cost benefit and preservation of reusable (RU) ureteroscope represent the main arguments for their adoption (11). Newer generation models are now available, and a recent meta-analysis of clinical studies reveal a non-inferior status regarding outcomes such as stone free rate (SFR) and complication burden when compared to RU scopes (12). The trajectory of their uptake appears likely to rise further owing to favourable physical properties. This includes ergonomic advantages such as lighter weight (some models are less than 100 g including cable) and novel modifications such as left and right-handed versions. Mixed conclusions have been put forward regarding the true cost efficiency of SU models and the reason for this is largely due to the different inputs used for the calculations such as the average life cycle of RU ureteroscope (range: 8–29 procedures) and the individual contract for repair costs between hospitals and suppliers (13). A proposed disadvantage of SU models has been the generally larger outer diameter sizes compared to RU scopes and more specifically, fibre-optic models. This has the potential to lead to sequalae such as lower rates of success at overcoming the ureteral orifice and a narrower space between the scope and the ureteral wall, which may result in a poor irrigation outflow and consequently increases the risk of high intrarenal pressure. Also, a larger scope calibre can result in the need for larger sized UAS. While the newer generation models are slimmer, they do not yet match the dimensions of RU alternatives such as the Olympus P7, which has a tapered 4.9Fr tip. The durability of SU ureteroscopes for surgeries of longer duration has also been questioned, given the issue of sudden image loss (14). To this end, a recent analysis of national registry of device failures associated with SU ureteroscopes found that image loss accounted for more than 75% of reported problems associated with their clinical use (15).

### The optimal laser source: holmium:yttrium-aluminium-garnet vs. thulium fiber laser vs. pulsed thulium:yttrium-aluminium-garnet

Holmium:yttrium-aluminium-garnet (Ho:YAG) is the current standard when performing URS and stone lithotripsy. This has been the case for over 30 years and while alternatives have been introduced, none of these were able to demonstrate superiority in the clinical setting (3). Thulium fiber laser (TFL), that also has a pulsed action, is arguably the first alternative that has challenged the dominant status of Ho:YAG (16–18). Several clinical studies now support the superiority found in earlier pre-clinical studies (19, 20). This includes a randomised trial by Ulvik et al. that found significantly higher SFR for renal stones associated with TFL use as well as fewer intra-operative adverse events and shorter operative times (21). However, SFRs for ureteral stones were the same (100%) and this highlights that in many scenarios (e.g., uncomplicated distal ureteral stone), even a low power holmium laser machine is still sufficient. Of note, while it was a 60 W TFL machine and 30 W Ho:HAG machine, the power setting of 2,4 watts used for both, which is well within the range of the machines. Another recent randomised study by Haas et al. found no differences in SFRs between these two lasers, regardless of stone location (22). However, in that study, the stone burden was comparatively extremely low with mean lasering times of 2.7 min for the Ho:YAG and 3.6 min for the TFL, while the per protocol power analysis assumed 6 min differences between the two groups. Additionally, follow up imaging was ultrasound (US) and plain x-ray (XR) rather than computed tomography (CT). That study also supports that any low power pulsed laser may be adequate when facing small stone burden.

Recently, the pulsed Thulium:YAG laser has been proposed as a further alternative to the Ho:YAG, with promising stone dusting proprieties (23). Two clinical studies are available to date and reveal the pTm:YAG as an efficient and safe laser for lithotripsy (24, 25).

No comparative studies evaluating the Ho:YAG against the TFL and pTm:YAG are available to date, therefore no clear recommendation can be made as to whether either to TFL or the pTm:YAG may become the new gold standard for lithotripsy.

### Basketing versus dusting

Regardless of type, laser has become an established energy source for stone lithotripsy. However, the laser strategies employed by surgeons do vary. More specifically, continued debate exists regarding whether the more traditional approach of fragment and basketing is superior to dusting (26). Although no difference between the two strategies was found in the EDGE study from North America, it is important to point out, though, that almost none of the patients had a CT scan for follow-up (27). A recent meta-analysis of 10 studies found no significant difference for SFR, re-treatment rate or complications (28). In that study, the most popular dusting settings were 0.2–0.5 J and 15–20 Hz (3–10 Watts). In addition to the predominant use of plain XR to assess SFR, none of the studies to date have employed TFL, which lends itself to dusting. Consensus is still lacking regarding what constitutes dust and definitions currently range between 200 and 400 μm sized particles (28, 29). In practical terms, it could be considered the particles of such size that can be aspirated through the working channel of the ureteroscope. Proponents for dusting may also argue that accessories such as the basket are becoming obsolete and adverse events can occur during their use (30). However, in a recent randomised trial by Yaghoubian et al., where lower pole stones were either displaced with a basket before lithotripsy or treated in site, patients in the former group achieved significantly higher SFR (95% vs. 74%, *p* = 0.003) (31). Arguably, basketing may remain as a primary choice according to the clinical scenario and personal preference of surgeons, but current trends have confirmed an emphasis towards dusting techniques (32).

### Ureteral access sheath

Application of UAS holds potential advantages including reduction of intra-renal pressure and subsequent infectious complications, as well as improved irrigation and endoscopic vision accordingly. A statewide study of over 5,000 URS procedures by Meier et al., revealed that use of UAS among surgeons varied between 1.8% and 96% (33). That particular study highlighted not only the contrasting personal preferences of surgeons towards this accessory, but also potential limitations as the authors found a significantly increased likelihood of increased emergency department (ED) presentation and hospitalization associated with use of UAS. It is indeed these concerns regarding the adverse events why UAS hold a controversial status. This includes intra-operative complications such as ureteral perforation and late sequelae such as ureteral stricture formation (34). Improved dusting capabilities that are enabled with newer laser platforms arguably reduce the need for the relay of fragments out of the kidney via UAS. At the same time, it can be argued that the smaller dimensions of newer ureteroscopes allow for smaller diameter UAS to be used and hence, there is a reduced risk of associated ureteral injury. Of note, using a smaller diameter UAS, diminishes the effect on intrarenal pressures (35). Furthermore, the smaller sized laser fibers available with TFL, allow for more irrigation to be delivered via the working channel and therefore improved vision. There are numerous individual studies where the findings either support or disfavour UAS, but when the literature is reviewed as a whole, it seems the data is still inconclusive (36).

### Safety guidewire in routine URS

A safety guidewire (SGW) is a guidewire that is introduced during initial cystoscopy and kept in the ureter adjacent to the ureteroscope throughout the procedure. In the advent of the ureteroscopic era, the SGW was a valuable tool aiding in maneuvering the large diameter endoscope up to the ureter. Since then, despite miniaturisation of the ureteroscopes, the SGW has been considered a formal requirement when performing URS by many experts. They offer an exit strategy when faced with unforeseen intra-operative complications such as ureteral perforation. While there is agreement regarding their merits in scenarios such as difficult ureteric anatomy or heavily impacted stone with a clear risk for worsening any ureteral damage, debate exists regarding whether they should be mandatory in URS determined to be routine or uncomplicated. That is because their employment can hinder the surgeon in terms of advancing up the ureter alongside a SGW. In fact, a randomised trial demonstrated that the forces needed to introduce and retract the ureteroscope in the ureter increased more than 100% when a SGW was in place compared to when omitted (37). In a comparative trial with 500 URS with SGW and 500 URS without SGW, the same group also studied the proposed benefits of using an SGW, that is increased success of entering the ureteral orifice, easier maneuvering up the ureter in terms of reaching the stone level, and most importantly preserve the ability to place a stent at the end of the procedure (38). The study showed no difference between the groups in any of the suggested benefits of using an SGW, and the authors concluded that routine use of SGW during URS should not be mandatory. Opponents to this might argue that one does not know if a case will really be a routine operation until it is too late. The European Association of Urology (EAU) guidelines do recommend their use, however the level of evidence to support this is only expert opinion (39). Interestingly, all studies that have evaluated the topic have revealed no increased association with complications, although these studies were arguably underpowered, since the event of a SGW that is going to be used for safety is extremely rare. Moreover, this includes two randomised trials that not only support this finding but also reveal longer operation times associated with use of a SGW (40–42).

### Fluoroscopy free URS

There is agreement among surgeons to follow the “as low as reasonably achievable” (ALARA) principles regarding intra-operative use of fluoroscopy (43, 44). Active measures can be taken by the surgeon including use of pulsed fluoroscopy and image collimation (45). Several studies have sought to determine an association of higher dosages when control of the C-arm is primarily by surgeon or an assistant/radiation technologist, and overall there appears to be no difference (42). This includes a recent randomised trial by Kokorowski et al. (46). There has been more attention recently towards zero use of fluoroscopy. Adaptations can be made to the standard technique such as marking length on ureteroscope once positioned in the pelvic ureteric junction to facilitate insertion of UAS. Here too, ureteral stents are inserted using tactile feedback rather than fluoroscopy control. Use of real time ultrasound has also been presented as a tool (47). There is a number of cohort studies reporting this approach in large patient samples and without an increased complication burden. However, these are usually highly experienced, single surgeon series and more difficult patient groups such as urinary diversions are usually excluded (48). Of note, almost all case reports on unintentional DJ-stent insertion into large vessels report the lack of fluoroscopy during the intervention (49). By analogy, fluoroscopy free ureteroscopy might cause rare, but disastrous events. A simple and effective method of reducing fluoroscopy time during URS is to increase awareness of the topic, and the surgeon should ask him- or herself whether there really is a need for fluoroscopy *every time* the pedal is activated. The most recent systematic review and meta-analysis on this topic including 24 studies, among which 12 have been randomized, revealed that no significant differences exist in stone-free rates, length of stay and operative time between fluoroscopy-free and fluoroscopy-guided procedures, with complications been higher in the fluoroscopy-guided group (50). These findings were similar when URS and PCNL was analysed separately, while the overall conversion from fluoroscopy-free to fluoroscopy-less procedure was 2.84%.

### Imaging for assessment and follow up

Beyond the surgery itself, there is debate and differing practice patterns regarding both work up and follow up of urolithiasis patients. Imaging type and timing form a large part of this debate. CT delivers the highest sensitivity and specificity for diagnosing urolithiasis compared to alternatives such as US and plain XR. Low dose versions also allow the dosage to be reduced further while still providing necessary information. Such are the merits of CT for assessing stone burden that some scientific journals have started requiring it as a standard for submission (51). This does present difficulties in less resource rich areas as well as special populations such as children, in addition to the argument about higher radiation dosages. Related to imaging type is how stone size is reported. The current standard in guidelines is based on the maximal diameter (39). However, two stones with the same maximal dimensions can in fact have quite different overall sizes if the volume is measured (52). Measurement of stone volume has therefore been recommended as a means to give more accurate assessment of stone burden. From a research perspective, it could also allow for more accurate evaluation of laser energy consumption (Joules/mm^3^), e.g., when comparing Ho:YAG and TFL (53). However, adopting stone volume is not without problems as there several methods to calculate it. Even when the formula has been decided upon, manual measurement of three dimensions increases the risk for inaccuracy for each one that is subsequently multiplied. It can also be a relatively time intensive process. Automated calculation with software represents one solution to this but these are not yet available as an integrated tool within hospital systems and can be expensive. Exporting patient sensitive information also presents privacy concerns.

## Future directions

For all these contested topics, more randomised trials will help guide future clinical practice. Part of the reason why it can be so challenging to compare outcomes across different studies, regardless of their type, is the heterogeneity in reporting that is present. Implementation of reporting tools such as the Adult-Ureteroscopy (A-URS) Checklist could serve to help address this (54). This tool offers an overview of suggested study details and parameters to be reported.

## Strengths and limitations

This narrative review has certain limitations to acknowledge. Firstly, the literature search was non-systematic and therefore not unabridged. A large number of the studies reviewed were of a low level of evidence including expert opinion. To this end, the conclusions need to be considered in light of this. However, this review offers the reader an overview of the core issues surrounding each topic. The findings in this review can therefore serve as a useful aid to the time pressured clinician.

## Conclusion

In the field of URS, there are many controversies. Technological advances allow for improved patient outcomes but adoption of a particular technique over an another is largely based on surgeon preference. Surgeons are encouraged to explore and understand the advantages and disadvantages of each of these so as to enable a tailored approach for their patients and practice as a whole.
