# Supplementary material for: Controversies in ureteroscopy: lasers, scopes, ureteral access sheaths, practice patterns and beyond
Source: Front Surg. 2023 Sep 13;10:1274583. doi: 10.3389/fsurg.2023.1274583 (PMC10533910; doi:10.3389/fsurg.2023.1274583)
Supplement: Supplementary file 1 [file Table1.docx]

| **For** | **Against** | **Summary** |
| --- | --- | --- |
| **Single use ureteroscopes** | | |
| Outcomes reported are non-inferior  Always sterile (potential to reduce contamination risk) Always new (without traces of instrument wear)  Advantages in cases at high risk for scope damage  Useful for resident training  Better ergonomics (lower weight) No need of a dedicated sterilization process  Future potential for customized scopes (e.g. shorter scopes for female patients, varying working channel position, etc…) | Environmental burden not fully known  Less durability for long cases (sudden image loss)  Still a relatively limited number of clinical studies (especially lacking randomised controlled trials)  Cost burden difficult to determine  Tip sizes still do not match certain reusable models | Centres should perform micro-cost analysis locally to determine cost efficiency. Use is set to continue to increase. Quality of scopes continuously improving. Still more data needed. |
| **Holmium: YAG Laser** | | |
| Clinical properties are well studied and understood. Low power still effective for ureteral and kidney stones. High power machines have no clear advantage, except pulse modulation which may reduce retropulsion.  Versatility for other applications such as laser enucleation of the prostate. | High power machines incur higher costs and risks associated with high irrigation temperature rises  Few clinical studies support a true clinical benefit of pulse modulation techniques such as Moses technology. | Use is well established. Low power can be applied for use with uncomplicated ureteral and kidney stones and achieve similar results to high power and TFL. |
| **Thulium Fiber Laser** | | |
| A limited amount of studies have showed advantages such as faster operating time, better view, less bleeding and improved stone free rates compared to Ho:YAG  Available in smaller fibre size  Advantageous coagulation proprieties for mixed applications such as incision of a stone-bearing diverticular calyx | Optimal settings still not known  Concerns raised regarding flash and carbonisation intra-operatively.  Cost relatively high (but might be outweighed by shorter operative times) | Increasing number of clinical studies that show a benefit over Holmium, especially low power. Advantageous if wish to dust. |
| **Pulsed Thulium:YAG Laser** | | |
| Promising dusting proprieties according to preliminary in vivo studies  Versatility for other applications such as laser enucleation of the prostate | Only two studies have evaluated this laser in a clinical scenario until today. |  |
| **Stone dusting** | | |
| Improved laser systems allow for effective dusting  Less auxiliary equipment needed such as baskets and therefore lower risk of injury  Multiple stone dust aspiration devices in development and soon to become available for clinical routine | Consensus on dust definitions still not achieved. Mostly relies on having a new generation laser that has required settings  Dust is not all necessarily passing spontaneously when left in kidney | Both methods are effective and there is a lack of published data to support superiority of particular technique. More studies including stone dust suction devices and using CT as follow up imaging will help determine which is superior. |
| **Ureteral access sheath** | | |
| Improved irrigation flow and vision  Maintain lower intra-renal pressure when large diameter sheaths are used  Ease of multiple scope passages for basketing of numerous stone fragments | Higher stricture rate at long term  More early re-admissions and complications  Avoidable additional costs  Potential damage to ureteroscope | There are a wide number of studies with conflicting results. The data is still inconclusive. |
| **Safety guidewire for routine URS** | | |
| Allows ureteral stent to be placed if injury sustained | Need for more equipment  Many guidewires / patients need to be treated for the benefit of one event /patient when the guidewire will truly save the situation | It is extremely rare that a safety guidewire will be important, and therefore it would be very difficult to set up a study that has a sufficient power to proven/revoke the necessity of a GW  Use is ultimately determined by personal preference and training background. |
| **Fluoroscopy free ureteroscopy** | | |
| Reduce radiation exposure to patient, surgeon and operating staff. | Data is limited to single surgeon series  Only suitable for experts and uncomplicated cases  Almost all case reports on unintentional DJ-stent insertion into large vessels report the lack of fluoroscopy during the intervention. By analogy, fluoroscopy free ureteroscopy might cause rare, but disastrous events. | Studies are limited to a few cohort studies from highly experienced surgeons only. Application should be judicious and with relevant experience. |

**Table 1.** Summary of controversial topics
